# Endoscopic incision for removal of an impacted appendiceal fecalith

**DOI:** 10.1055/a-2408-9833

**Published:** 2024-09-19

**Authors:** Jingjing Yao, Qing Shao, Jing Wang, Xin Wei, Jindong Fu

**Affiliations:** 1 549615 Gastroenterology, Rizhao Peopleʼs Hospital, Rizhao, China

A 69-year-old man was admitted to our hospital with 1-week history of intermittent lower abdominal pain. He had a history of subtotal gastrectomy performed 6 years prior for gastric carcinoma. An abdominal computed tomography scan revealed a localized high-density area in the appendix ( Fig. 1 ). Colonoscopy showed a significantly protruding appendix with an impacted fecalith, resulting in marked dilation of the appendiceal orifice ( Fig. 2 ). This appearance resembled that of impacted stones in the duodenal papilla.

**Fig. 1:**
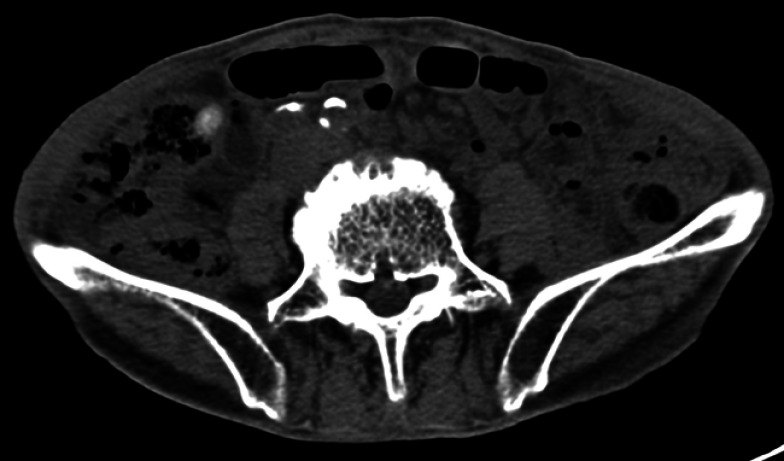
Abdominal computed tomography scan demonstrating a localized high-density area in the appendix.

**Fig. 2:**
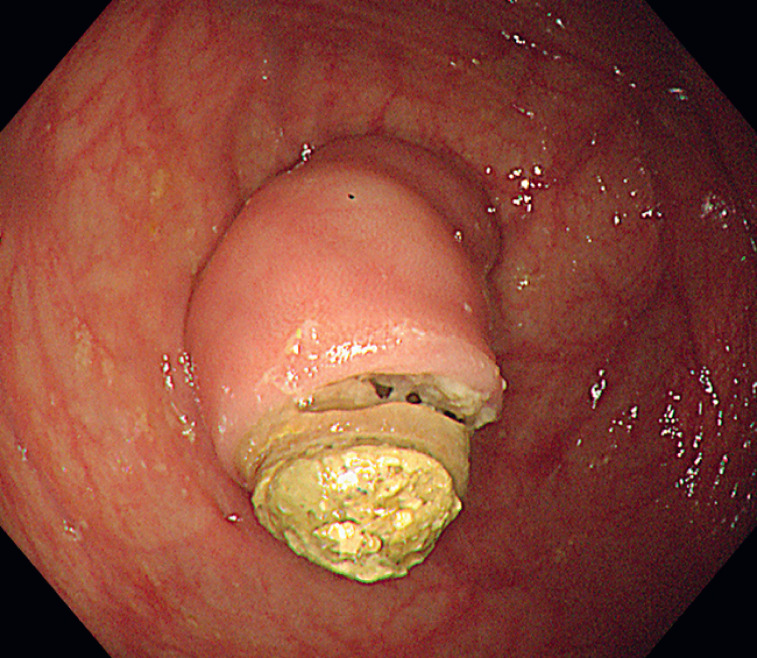
Endoscopic view of a significantly protruding appendix with an impacted fecalith.

After obtaining informed consent, we performed endoscopic removal of fecalith ( Video 1 ). A 1.5-mm Goldknife (Micro-tech, Nanjing, China) was inserted into the appendiceal lumen, and a full-thickness incision was made from the dilated appendiceal orifice to the base of the protrusion, without requiring submucosal injection. Then, the fecalith was fully exposed and extruded into the colonic lumen with the aid of the Goldknife ( Fig. 3 ). Following the removal, the appendiceal lumen was irrigated with water, and no bleeding or perforation was observed. The procedure was completed without immediate complications, and the incision was left open ( Fig. 4 ). The patient was kept fasting for 6 hours after the procedure. His abdominal symptoms resolved, and no postoperative complications were noted. He was discharged 2 days after the procedure.

**Fig. 3:**
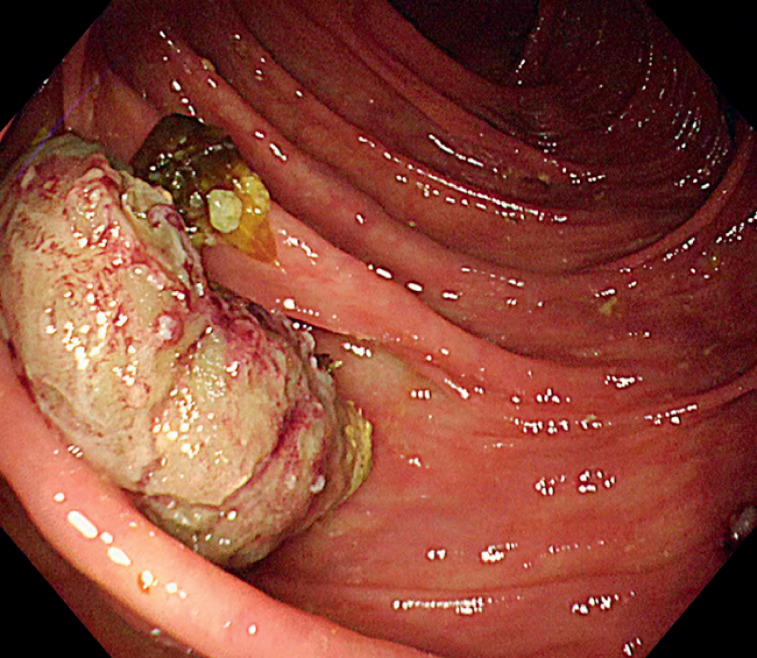
Endoscopic view of the fecalith after extrusion into the colonic lumen.

**Fig. 4:**
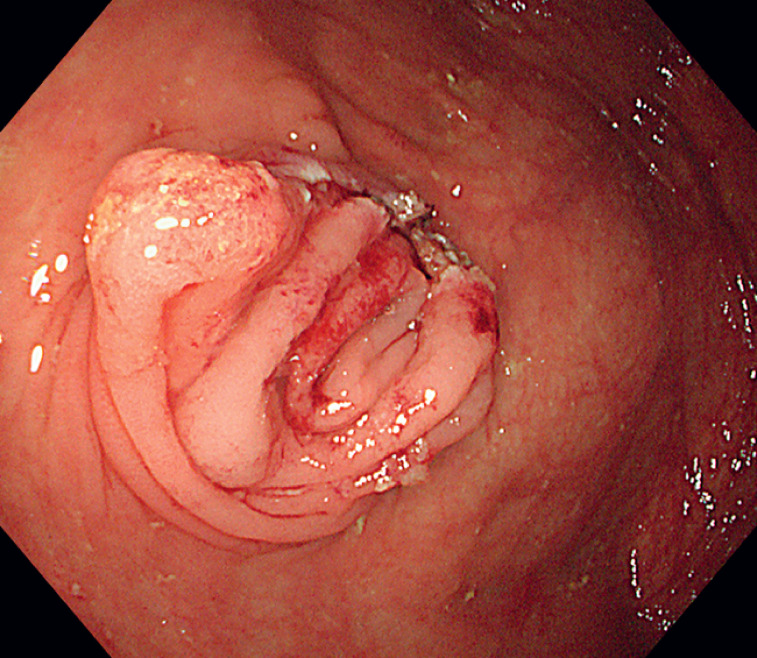
Endoscopic view of the appendiceal incision left open for healing after removal of the fecalith.

Endoscopic removal of impacted fecalith from the appendix in a 69-year-old man with 1-week history of intermittent lower abdominal pain.Video 1

Appendiceal fecaliths are commonly implicated in acute appendicitis due to their obstructive potential within the appendiceal lumen. Traditional management typically requires surgical intervention 1 2 . However, in the present case, endoscopic incision was successfully used to facilitate the removal of an impacted fecalith. Our experience suggests that endoscopic incision can provide a feasible and less invasive treatment option for patients with impacted fecaliths, potentially reducing surgical risks and expediting recovery.

Endoscopy_UCTN_Code_TTT_1AQ_2AF

## Notes

Endoscopy E-Videos https://eref.thieme.de/e-videos
*E-Videos* is an open access online section of the journal *Endoscopy* , reporting on interesting cases and new techniques in gastroenterological endoscopy. All papers include a high-quality video and are published with a Creative Commons CC-BY license. Endoscopy E-Videos qualify for HINARI discounts and waivers and eligibility is automatically checked during the submission process. We grant 100% waivers to articles whose corresponding authors are based in Group A countries and 50% waivers to those who are based in Group B countries as classified by Research4Life (see: https://www.research4life.org/access/eligibility/ ). This section has its own submission website at https://mc.manuscriptcentral.com/e-videos .
